# Systems, Properties, Surface Modification and Applications of Biodegradable Magnesium-Based Alloys: A Review

**DOI:** 10.3390/ma15145031

**Published:** 2022-07-20

**Authors:** Junxiu Chen, Yu Xu, Sharafadeen Kunle Kolawole, Jianhua Wang, Xuping Su, Lili Tan, Ke Yang

**Affiliations:** 1 Key Laboratory of Materials Surface Science and Technology of Jiangsu Province, Changzhou University, Changzhou 213164, China; xuyuall@163.com (Y.X.); wangjh@cczu.edu.cn (J.W.); sxping@cczu.edu.cn (X.S.); 2 Jiangsu Collaborative Innovation Center of Photovoltaic Science and Engineering, Changzhou University, Changzhou 213164, China; 3 Haian High Technology Industry Research Center, Changzhou University, Nantong 226600, China; 4 Mechanical Engineering Department, School of Engineering and Technology, Federal Polytechnic, Offa P.M.B 420, Nigeria; eskaypumping@yahoo.co.uk; 5 Institute of Metal Research, Chinese Academy of Sciences, Shenyang 110016, China; lltan@imr.ac.cn

**Keywords:** biodegradable Mg alloy, alloying, surface modification, clinical application

## Abstract

In recent years, biodegradable magnesium (Mg) alloys have attracted the attention of many researchers due to their mechanical properties, excellent biocompatibility and unique biodegradability. Many Mg alloy implants have been successfully applied in clinical medicine, and they are considered to be promising biological materials. In this article, we review the latest research progress in biodegradable Mg alloys, including research on high-performance Mg alloys, bioactive coatings and actual or potential clinical applications of Mg alloys. Finally, we review the research and development direction of biodegradable Mg alloys. This article has a guiding significance for future development and application of high-performance biodegradable Mg alloys, promoting the future advancement of the magnesium alloy research field, especially in biomedicine.

## 1. Introduction

Since the beginning of this century, with the development of science and technology, biodegradable metals have also developed rapidly and attracted increasing attention. Among biodegradable metals, magnesium (Mg) alloy is the most representative. Mg alloys possess unique properties, such as low density, low elastic modulus and good biocompatibility [1]. They have been proven to be a safe and effective medical implant materials by many studies. Mg ions released from the alloy showed good biological activity [2]. When an Mg alloy is implanted into the human body, it is degraded and absorbed [3,4]. Excessive Mg ions are also excreted by the metabolism of human body, and Mg alloy implants do not require secondary operations, owing to their biodegradation, which considerably reduces the incidence rate and risk of trauma. However, the rapid degradation rate of Mg alloys remains the main obstacle to their widespread application [5,6]. Alloying and surface modification are the most commonly used methods to improve the corrosion resistance and bioactivity of Mg alloys. Moreover, many important achievements have been obtained in both alloy design and surface modification. New high-performance Mg alloys have been explored, including Mg-Zn-based alloys, Mg-RE (rare earth)-based alloys, Mg-Li-based alloys, etc. In addition, some highly bioactive coatings have been developed, including microarc oxidation coating, polymer coating, composite coating, etc. With ongoing research, an increasing number of Mg alloy implants are approaching the stage of clinical applications, such as stents, screws, structures, etc. These developments will be reviewed in the following sections.

## 2. Research on High Performance Biodegradable Mg Alloys

Due to the relatively poor mechanical properties of pure Mg, its clinical application is limited. Many studies have been carried out to improve its mechanical properties and corrosion resistance by alloying. At present, the studied high-performance Mg alloys are mainly Mg-Zn based alloys, Mg-Ca based alloys, Mg-Li based alloys, Mg-Cu based alloys and Mg-RE based alloys.

### 2.1. Mg-Zn-Based Alloys

Zn is a trace element in the human body necessary to maintain physiological function, with the characteristics of osteogenesis and antibacterial ability [7,8], and is mainly stored in bones and muscles (>85%). The Zn^2+^ produced during the degradation of Mg-Zn alloy is excreted by the body’s metabolism and does not accumulate in the organs [9]. Both Mg and Zn have a hexagonal, close-packed (HCP) crystal structure. The density of Mg-Zn alloy increases with increasing Zn content. Mg and Zn can combine to form an MgZn_2_ metallic compound. After hot extrusion and aging treatment, Mg-5Zn alloy was found to form a more uniform and stable Mg(OH)_2_ protective corrosion product layer in Ringer’s solution, with considerably improved corrosion resistance [10]. Dong et al. [11] found that the corrosion rate of Mg-4.1Zn alloy decreased from 2.3 ± 0.9 mm/y (mm/year) to 0.7 ± 0.1 mm/y after immersion in simulated body fluid for 28 days. Zn has a strengthening effect in Mg matrix; for instance, the yield strength and Young’s modulus of Mg-4.1Zn alloy are about three times those of pure Mg.

### 2.2. Mg-Ca-Based Alloys

Ca is an essential dietary element in the human body and the main component of bones, i.e., most calcium is stored in bones. Ca, as an alloying element, has a grain-refining effect. Xia et al. [12] proved that adding Ca into an Mg matrix reduced the grain size and corrosion rate, with grain sizes of Mg-xCa (x = 0.5, 0.8, and 2.0) of 70–100, 50–70 and 20–50 μm, respectively. The higher the Ca content, the smaller the grain size. By evaluating the sample volume and the rate of hydrogen evolution, it was found that after immersion in 3.5% NaCl solution for 100 h, Mg-2.0Ca had the lowest corrosion rate of 0.94 mm/y. Parfenov et al. [13] also showed that refining Mg-1Ca grains by high-pressure torsion treatment and annealing improved the corrosion resistance of the nanostructured alloy in Ringer’s solution. When the grain size ranged from 0.9 to 1.3 μm, i.e., ultrafine grains (UFG), the Mg-1Ca alloy had the best corrosion resistance, with a constant corrosion rate at −0.052 g·dm^−2^·d. Geanta et al. [14] found that Mg-Ca-Zn produced by a suspension induction smelting furnace has a uniform microstructure, and hardness test results also showed good mechanical properties. Chen et al. [15] showed that Mg-0.8Ca-5Zn-1.5Ag (ZQ71) alloy had better biocompatibility and higher osteogenic activity than Mg-0.8Ca (ZQ) alloy. With the addition of Zn and Ag, the corrosion resistance of ZQ71 alloy was significantly improved. The lower degradation rate and the stimulation of metal ions caused the ZQ71 alloy exhibit higher osteogenic activity. Yurchenko et al. [16] also found that after multiaxial deformation (MAD), Mg-0.8%Ca underwent grain refinement, with an grain average size of 2.1 μm. The ultimate tensile strength of multiaxially deformed Mg-0.8%Ca following annealing was increased from 78 MPa to 308 MPa, and the elongation at break was increased from 4.1% to 7.2%. Istrate et al. [17] found that Mg-0.9Ca-1.2Zr alloy obtained by argon smelting formed eutectic compound Mg_2_Ca at the alpha-Mg grain boundary, which improved the corrosion resistance of the alloy. Geantă et al. [18] prepared Mg-Ca series alloys with a controllable argon suspension device, and the experimental results showed that only when the content of Ca was less than 2.5% was there a trend of formation and proliferation of living cells on the alloy surface.

### 2.3. Mg-Li-Based Alloys

Li is beneficial in terms of enhancing both the strength and corrosion resistance of Mg alloys. The crystal structure of Mg is hexagonal and close-packed (HCP), which limits its plastic deformation. Thus, the addition of Li can change the crystal structure of Mg from an HCP structure to a body-centered, cubic structure, which enhances the plasticity of Mg alloys [19]. Li et al. [20] showed that among extruded Mg-xLi (x = 1, 2, 5 wt%) alloys, Mg-5Li has the best corrosion resistance, and Mg-1Li has the worst corrosion resistance. With increased Li content, the crystal structure of Mg-xLi alloy remained constant with change from the basal structure of Mg-1Li alloy to the prismatic structure of Mg-5Li alloy. This considerably improved the corrosion resistance of Mg-5Li in 1M NaCl solution. Chen et al. [21] found that the extruded Mg-14Li alloy exhibited uniform and slow corrosion behavior in a simulated body fluid (minimum essential medium, MEM). In the MEM solution, a 25 μm thick dense film composed of lithium carbonate (Li_2_CO_3_) and calcium hydroxide (Ca(OH)_2_) was formed on the surface of Mg-14Li, which suppressed the rate of hydrogen evolution. Zhang et al. [22] found that the ultimate tensile strength of Mg-14Li-0.5Ni after homogenization was increased to 127MPa. Uniform elongation increased from 9% to 15%, and elongation at break increased from 30% to 50%. Further study also showed that the ultimate tensile strength of the Mg-Li alloy (LA103Z) after friction-stir processing was significantly increased from 178 MPa to 268 MPa, i.e., the mechanical properties were significantly improved [23].

### 2.4. Mg-Cu-Based Alloys

Cu is an important trace element for many enzymes in the human body, and the Cu^2+^ released from Mg-Cu alloy during the degradation could enhance the antibacterial ability and destroy bacterial cells [24]. As the Mg-Cu alloy degraded, the activity of Staphylococcus aureus was reduced by both high alkalinity and copper ion release, demonstrating its excellent antibacterial properties. However, the degradation rate of Mg-Cu alloy is usually fast, although a well-tailored heat treatment operation and suitable plastic deformation can reduce its degradation rate. Yan et al. [25] found that solution treatment likewise enhanced the corrosion resistance of Mg-0.1Cu alloy. They found that the corrosion rate of solution-treated Mg-0.1Cu alloy in Hanks’ solution was 1.8 mm/year, whereas that of the as-cast alloy was very high—45 mm/year, which is 25 times of that with solution treatment. Zhang et al. [26] showed that the biodegradation rate of the Mg-0.2Cu alloy treated by surface mechanical grinding was significantly reduced, and the alloy exhibited a good antibacterial effect. Further studies showed that the microstructure had an important influence on the mechanical properties and degradation rate of Mg-xCu (x = 0.2, 0.5) alloys. After extrusion and solution treatment, Mg-0.2Cu exhibited the best mechanical properties, with a yield strength reaching 209 MPa and an ultimate tensile strength of 264 MPa [27]. Li et al. [28] also found that Mg-0.25Cu alloy had considerable potential to be used for the treatment of osteomyelitis. However, in Mg-xCu (x = 0.05, 0.1, 0.25) alloys [28], the ion release of Mg-0.25Cu was much faster than that of the other two alloys, indicating that the addition of excessive Cu negatively affected corrosion resistance. In the future work, corrosion resistance of Mg-Cu alloy should be further reduced in order to make good use of its excellent antibacterial property and broaden its applications.

### 2.5. Mg-RE-Based Alloys

**Figure 1:**
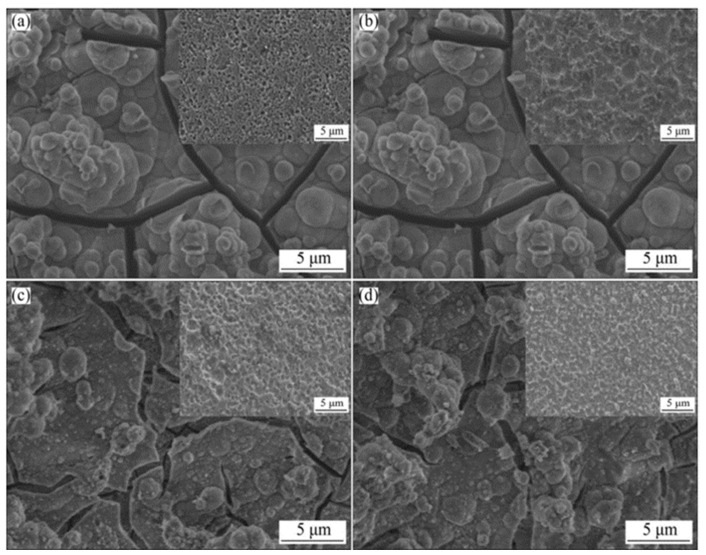
Surface morphologies of samples soaked in Hank’s solution with inserted corresponding surface images after removing corrosion products: (**a**) extrusion; (**b**) 325-30; (**c**) 350-5; (**d**) 450-33 (reprinted from Ref. [37] with permission).

Due to the presence of rare earth metal elements, Mg-RE-based alloys have high mechanical properties and corrosion resistance [9,29]. Researchers have developed some high-performance Mg-RE-based alloys, such as Mg-Nd-Zn-Zr, Mg-Gd-Dy-Zr, WE43, etc. The addition of yttrium (Y), for instance, can improve the atomic slip and ductility of magnesium alloy [30,31]. Gd, on the other hand, can remove impurities (such as Fe, Ni and Cu) in Mg alloy and reduce the corrosion rate [32,33]. Furthermore, Nd has the ability to refine grains. Therefore, adding Nd into Mg alloy can form an Mg_12_Nd phase, which could reduce the potential difference with the Mg matrix and ultimately improve the corrosion resistance of Mg alloy [34]. Li et al. [35] found that the ultimate tensile strength of Mg-1.2Gd-0.6Dy-0.2Zr alloy was increased by about 13.2% and the elongation was increased by about 100% after hot extrusion. The addition of rare earth metal elements promotes the dynamic recrystallization of Mg alloys and increases tensile strength without loss of elongation. Wang et al. [36] found that both Mg-10La and Mg-20Ce alloys prepared by vertical twin roll casting (TRC) technology had better corrosion resistance than AZ31 alloy. Observation of FE-SEM images showed that the microstructure of AZ31 alloy was composed of inhomogeneous dendrites, whereas that of Mg-RE alloy was amorphous. Electrochemical test results showed that Mg-RE alloys with amorphous structure had better corrosion resistance. After implantation in the femur of animals, more new bone tissues were found around the Mg-RE implant. Tian et al. [37] found that the corrosion rate of Mg-Nd-Zn-Zr Mg alloy decreased after annealing at higher temperatures for shorter times. The annealing parameters for the alloy wires were denoted as 325-30, 350-5 and 450-3 for 30 min at 325 °C and 5 min at 350 °C and 3 min at 450 °C, and the samples were then immersed in Hank’s solution at 37 °C. It was found (as shown Figure 1) that the corrosion pit of sample 325-30 was the largest, and the corrosion pit of sample 450-3 was slightly smaller than that of sample 350-5, indicating that its corrosion resistance was the best. Table 1 shows the mechanical properties and corrosion resistances of some typical Mg-RE alloys [32,33,34,35,36]. Most Mg-RE alloys have excellent mechanical properties and corrosion resistances; however, when rare earth metal elements are selected, focus should be on the cytotoxicity of the elements and their impact on the function of the human body.

**Figure 2:**
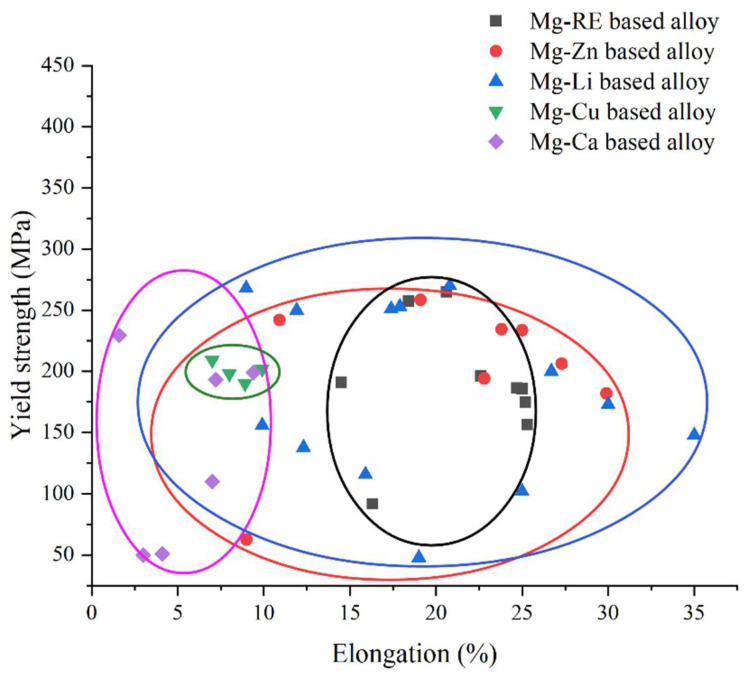
Ultimate yield strength and elongation of common magnesium-based alloys. Mg-RE-based alloys include cast, extruded, extruded + heat-treated Mg-4Y-2Er-2Zn-0.6Zr [43], annealed Mg-2.1Nd-0.2Zn-0.5Zr [37] and extruded Mg-4Gd-0.5Zr-xZn (x = 0, 0.2, 0.5, 1, 1.5) [44]; Mg-Zn-based alloys include extruded Mg-Zn-Mn-Sn-0.2Dy [45], homogenized Mg-2Zn-1Gd-0.4Mn-0.1Sr (ZGMS), extruded (at 320 °C and 360 °C) Mg-2Zn-1Gd-0.4Mn-0.1Sr (ZGMS) [46] and extruded (at 320 °C, 340 °C, 380 °C and 380 °C) Mg-Zn-Gd-Y-Zr [47]; Mg-Li-based alloys include room-temperature extruded and high-temperature extruded Mg-7Li-2Al-1.5Sn [48], friction-stir-processed Mg-9.98Li-3Al-2.81Zn [23], thermomechanically processed Mg-8Li-3Al-0.4Y [19], cast Mg-11Li (L11), Mg-11Li-3Zn (LZ113), Mg-11Li-6Zn (LZ116) [49], and cast and extruded Mg-8Li-3Al-xSn (x = 1, 2, 3) [50]; Mg-Cu-based alloys include extruded and solid-solution Mg-xCu (x = 0.2, 0.5) [27]; Mg-Ca-based alloys include cast, annealed, cast + MAD, annealed + MAD Mg-0.8Ca [16], equal-channel-extruded (ECAP) Mg-1Ca [51] and high-pressure torsion-treated Mg-1Ca [52].

In order to better study the mechanical properties of biodegradable Mg alloys, Figure 2 summarizes the yield strength and elongation of the above-mentioned representative biodegradable Mg-based alloys, including Mg-RE-based alloys [37,43,44], Mg-Zn-based alloys [45,46,47], Mg-Li-based alloys [19,23,48,49,50], Mg-Cu-based alloys [27] and Mg-Ca-based alloys [16,51,52]. Most Mg-RE-based alloys can ensure adequate tensile strength, as well as good elongation, and therefore have good application prospects. Traditionally, the overall mechanical properties of Mg-Cu-based alloys are poor, and the elongation is low at higher ultimate tensile strength. The addition of Li changes the crystal structure of the Mg matrix and enhances the plasticity of the Mg alloy. Therefore, Mg-Li-based alloys have good elongation, ensuring desirable tensile strength.

## 3. Development of Surface Modification of Biodegradable Mg Alloys

Degradability is both an advantage and a disadvantage of Mg alloys. Due to the rapid degradation of Mg alloys in vivo, localized corrosion can lead to stress concentration [53]. Hydrogen gas generated during the degradation process increases the local pH and impairs the growth of tissue cells around the implant, resulting in many uncontrollable factors during treatment [54]. Surface modification is an effective method to improve corrosion resistance and bioactivity of Mg alloys.

### 3.1. Microarc Oxidation Coating

**Figure 3:**
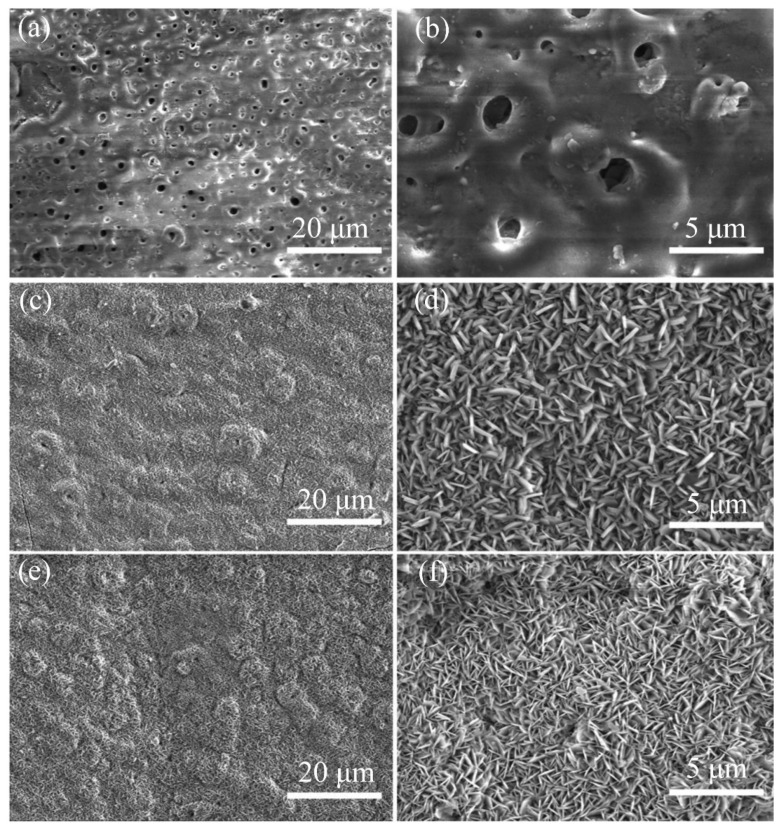
(**a**,**b**) SEM images of AZ91D alloy matrix after microarc oxidation. (**c**,**d**) SEM images of AZ91D alloy substrate after hydrothermal treatment. (**e**,**f**) SEM images of AZ91D (reprinted from Ref. [61] with permission).

Microarc oxidation (MAO) coating has good bonding force with the substrate, high hardness and excellent wear resistance [55]. MAO coating can effectively prevent the corroded metal ions from contacting the surface of Mg alloys, thereby improving the corrosion resistance of Mg alloys [56]. In the early stages of implantation, the protective oxide layer retards the rate of corrosion, and the reduced rate of hydrogen evolution promotes the growth of new bone around the implant [57]. Liu et al. [58] found that MAO coatings formed on HGM/Mg (hollow glass beads/magnesium) alloys provided effective corrosion protection for the substrate in Cl-containing solutions. Usually, micropores are easily corroded. Many studies on reduction of the diameter of micropores have been reported. Lin et al. [59] found that the addition of Li reduced the number of micropores and cracks on an MAO-coated pure Mg surface. Li-MAO coating was also reported to have better osteogenic ability according colorimetric method (MTT) and quantitative polymerase chain reaction (qPCR) analyses. Ma et al. [60] showed that MAO coating containing B_4_C/C particles prepared on an Mg-Li alloy had a better corrosion resistance. The addition of B_4_C/C particles resulted in a decrease in porosity in the MAO coating. The hardness of the Mg-Li alloy increased from 197.5 HV to 301.7 HV. Hydrothermal treatment is also a useful method to reduce the number of micropores. After MAO-treated AZ91D alloy was immersed into 2 mol/L KOH solution for hydrothermal treatment, its corrosion rate decreased from 9.62 mm/y to 3.54 × 10^−4^ mm/y [61]. Figure 3a,b shows SEM images of the porous structure formed by MAO. These micropores provided channels for ion migration and corrosion reaction during the MAO process, and corrosion resistance was degraded. Figure 3c,f shows SEM images after hydrothermal treatment, indicating that the surface structure of the sample became rough, and the micropores almost disappeared.

### 3.2. Polymer Coating

**Figure 4:**
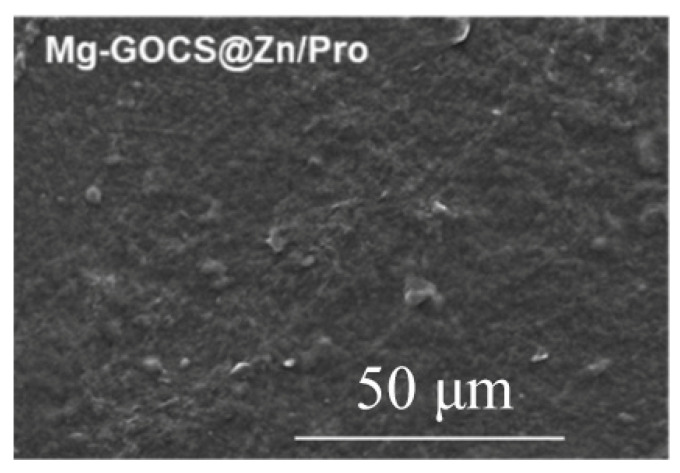
SEM of AZ31B alloy with multifunctional composite biocoating (reprinted from Ref. [64] with permission).

Polymer coatings usually exhibit good biocompatibility and biodegradability [62,63]. Pan et al. [64] showed that GOCS@Zn/Pro (graphene oxide (GO) chitosan (GOCS)) multifunctional composite biocoating effectively improved the corrosion resistance of AZ31B alloy to an annual corrosion rate of 0.05 mm/y due to a dense and complete protective layer formed on the surface of the alloy, which sufficiently covered the surface of the sample (Figure 4).

There are two main methods to fabricate polymer coatings on Mg alloys, namely dip coating and spin coating [65]. Wang et al. [66] reported that silane-coupling agent calcium hydrogen phosphate dihydrate (SCA/DCPD) coating prepared by dip-coating method resulted in AZ60 alloy with improved corrosion resistance, whereas silane-coupling agent calcium carbonate (SCA/CaCO_3_) coating prepared by biomimetic deposition method resulted in AZ60 alloy with improved biocompatibility. Al-Sherify et al. [67] reported that polymethyl methacrylate/hydroxyapatite (PMMA/HA) composite coating on AZ31 alloy prepared by spin-coating method effectively improved the corrosion resistance in Ringer’s solution. Due to the presence of the coating, the surface of Mg alloy had a uniform particle distribution, and the surface of the primary coating had significantly more cracks and micropores than the surface of the secondary coating. The hydrogen evolution from the alloy with two layers of PMMA/HA coating was 0.48 mL/cm^2^, which was about 2.32 mL/cm^2^ less than that from the bare AZ31 alloy. Further studies showed that polycaprolactone (PCL) polymer coating prepared on AZ31 alloy improved both corrosion resistance and bioactivity [68]. The total hydrogen volume from the bare AZ31 alloy sample in Hank’s solution was 7.92 ± 0.67 mL·cm^−2^, and that from the PCL-LS sample was 1.25 ± 0.28 mL·cm^−2^, i.e., the corrosion rate of the coating was reduced by about six times relative to that of the substrate. An in vitro fetal osteoblast (hFOBs) cell viability experiment showed that the survival rate of hFOB cells on the PCL-LS-coated samples after 5 days of culture was 86.1 ± 10.2%, indicating good biocompatibility.

### 3.3. Electrodeposition Coating

By depositing chemicals on the pores of the coating by calcification, electrodeposition and electrophoresis, a composite coating was formed, which improved both corrosion resistance and biological activity [69]. Saranya et al. [32] found that electrophoretic deposition of gadolinium oxide on Mg oxide increased cell proliferation and differentiation, as well as the expression of osteogenic genes. Compared with uncoated samples, the cell proliferation rate of coated samples was about 19% higher, and the total protein content was about 2.4 times higher. Pan et al. [70] found that aliphatic polycarbonate coating prepared by electrophoretic deposition and photo-crosslinking effectively improved the mechanical properties and corrosion resistance of AZ31 alloy. The alloy and pure Mg were soaked in SBF (37 °C) for 90 days. The measured Mg^2+^ content released from pure Mg was 670 mg/L, and the pH value of the sample solution was 8.5, whereas the Mg^2+^ content released from the coated sample was 440 mg/L. The Mg^2+^ content from the coated sample was much lower than that from pure Mg (36.1%). The composite coating can further improve the corrosion resistance of electrodeposited coating. Witecka et al. [71] reported that a coating of chitosan (CS) and bioactive glass (BG) particles on WE43 alloy by electrophoretic deposition (EPD) inhibited corrosion of the substrate. After soaking the samples in the osteogenic differentiation medium for 10 days, it was found that the mass loss of the composite coated samples was 8.9 ± 1.2 μg/mm^2^, whereas that of samples with only CS coating was 20.4 ± 4.4 μg/mm^2^. This shows that composite coating can considerably increase the corrosion resistance of Mg alloys. To date, layer-by-layer assembly coating has represented one of the main development directions. Askarnia et al. [72] found that HA/chitosan/graphene oxide (GO) ternary coating on AZ91D alloy by electrophoretic deposition resulted in good mechanical properties. When the GO content was 2 wt.%, the hardness of the composite coating increased from 40 ± 1.5 MPa of the substrate to 60 ± 3.12 MPa, and the corrosion rate decreased by 95%.

## 4. Application/Potential Application of Biodegradable Mg Alloys

With continuing research on biodegradable Mg alloys, an increasing number of Mg alloy implants have been developed, some of which have been used in clinical settings. At present, main types of clinical implants are coronary stents and bone fixation screws. Moreover, some implants, such as bone filling, sutures, dental-guide bone regeneration (GBR) film, etc., have been intensively developed.

### 4.1. Stents

Biodegradable Mg alloy coronary stents can support and stabilize the blood vessels in the early stage after implantation into the human body, preventing the rebound of blood vessels and the recurrence of atherosclerosis and helping to restore the original function of blood vessels [73]. In the later stage of implantation, with slow decomposition of the organism’s metabolism, no residue was found to be discharged from the body to reduce the risk of thrombosis. Researchers found that biodegradable Mg alloy stents have more advantages than other biodegradable metals [74].

**Figure 5:**
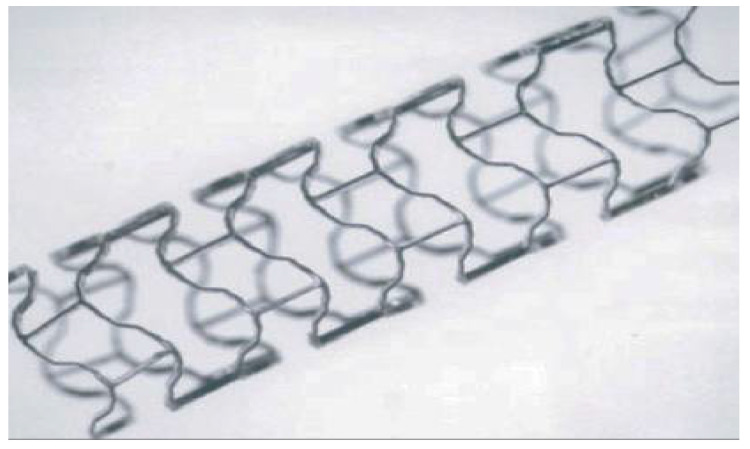
Magmaris BRS (Biotronik, Germany) (reprinted from Ref. [76] with permission).

In 2016, the DREAMS 2G (Magmaris) stent by Biotronik, a German company, obtained a CE certificate [75]. Figure 5 shows the Magmaris BRS from Biotronik [76], which is made of MgYREZR alloy and has been used in clinical settings. Haude et al. [77] found that the second-generation drug-eluting absorbable Mg alloy stent (DREAMS 2G) showed significant performance in blood vessels and was implanted in 123 patients with obstructive coronary artery disease. Only four (<4%) patients showed abnormalities. Angiography of the remaining patients identified vascular movement. In addition, optical coherence tomography did not detect any intraluminal mass or thrombosis. The target lesions implanted with DREAMS 2G had lower failure and revascularization rates. Therefore, this new biodegradable metal stent has considerable potential to replace absorbable polymer stents for the treatment of obstructive coronary artery disease, owing to its better load-bearing ability. Abellas-Sequeiros et al. [78] found that biodegradable Mg alloy stents (Magmaris) could be used for percutaneous coronary intervention (PCI) treatment. Magmaris was implanted in 45 patients without failure. The target lesion revascularization rate was 4.7%, which guaranteed the safety of Magmaris. During the 24-month observation period, the rate of thrombosis was 0%. In addition to clinical studies, some animal tests were also carried out by using other Mg alloys.

Further clinical studies found that magnesium-based bioabsorbable stents (MgBRS) had better effects on the treatment of patients with ST-segment elevation myocardial infarction. The results of this clinical trial were compared with those of permanent metal sirolimus-eluting stents (SES). The thrombosis rate in the MgBRS group was 1.4%, which was significantly lower than that in the SES group (2.6%). MgBRS was found to have a better vasodilatory response after 12 months. However, in the MgBRS group, more stents were lost in the cavity in the later stage [79].

Apart from clinical development, researchers also carried out some animal experiments using other Mg alloys to show the application feasibility. Xue et al. [75] found that tracheal stents made of Mg alloys (Mg-Nd-Zn-Zr alloy and four Mg-Ca-Zn alloys) could be used for patients with tracheal stenosis, exhibiting good feasibility in younger patients. In that study, five kinds of Mg alloys were studied. All five Mg alloys were successfully implanted into the trachea of New Zealand rabbits and degraded slowly in vivo, and there was no inflammatory response in the implanted part. Di et al. [80] demonstrated that Mg-4Zn-1Sr (ZJ41) alloy fabricated by semi-solid technique could be used to manufacture biodegradable ureteral stents, and in vivo experiments have also demonstrated its feasibility. A rheological solidification process was used to produce semisolid ZJ41 alloy, which resulted in finder alloy grains. This enhanced the corrosion resistance of the ZJ41 Mg alloy, and the entire stent degraded more uniformly. ZJ41 alloy stents were implanted into Guangxi Bama minipigs, and within 14 weeks of implantation, no inflammation or lesions were caused by the stents. Furthermore, the stent showed good antibacterial activity. The degradation time (14 weeks) of ZJ41 alloy stents in vivo meets the requirement of stents staying in the human body. The disadvantage of this experiment was that only one type of animal was studied, resulting in certain limitations.

### 4.2. Internal Bone Fixation

**Figure 6:**
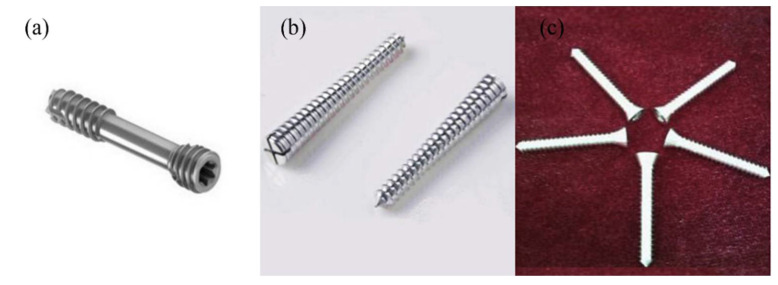
Biodegradable Mg screws that can be used clinically: (**a**) MAGNEZIX® CS screw (Syntellix AG, Hanover, Germany) (reprinted from Ref. [89] with permission), (**b**) bioabsorbable headless screw (K-METTM, U&I Corporation [90]) and (**c**) high-purity magnesium screws (Dongguan Eontec Co., Ltd., Dongguan, China) (reprinted from Ref. [91] with permission).

Screws are a common means of fracture fixation and are generally used in the fixation of bones, such as hallux valgus and wrist fractures [81]. The clinical requirements for screw materials are very strict. There should be a small difference in elastic modulus between the material of the implanted screw and the natural bone in order to reduce the stress shielding effect [82]. Implants should also have improved biological safety; otherwise, patients may present with hypersensitivity reactions to non-degradable implants, causing complications [83]. In the early stage, good mechanical properties and stability should be ensured to help the fracture heal. In the later stage, implants must have good biodegradability and be discharged through the human excretory system to reduce the risk of trauma to patients [84]. Biodegradable Mg alloy screws have begun to be used for bone fracture fixations [85]. In 2013, MAGNEZIX screws prepared by powder metallurgy (PM) from Syntellix AG in Germany obtained a CE certificate and entered the medical device market for the fixation of small bones and bone fragments [81,86]. The 3.2 mm diameter MAGNEZIX® CS is mainly used for hallux valgus surgery and scaphoid fractures [87]. In 2014, the Korea Food and Drug Administration approved the listing of Mg-Zn-Ca alloy nails for palmar bone fractures. In 2015, the RESOMET/K-MET screw developed by U&I Company in South Korea obtained a CE certificate [88]. In May 2020, a high-purity magnesium screw developed by Dongguan Eontec Co., Ltd. China obtained a CE certificate. Figure 6 shows the bone screws that have been used clinically [89,90,91]. Further clinical studies showed that Mg-based compression screws (MAGNEZIX^®^CS) had a significant effect on the treatment of bone fractures. A total of 29 patients were enrolled in the clinical trial, all of whom recovered without inflammation [92]. Jungesblut et al. [93] showed that Mg nails (MAGNEZIX) could be used to fix osteochondritis dissecans (OCD) lesions and displaced osteochondral fragments. All 19 patients implanted with MAGNEZIX were observed for 11 months. Of these patients, 12 healed completely. In one patient, the Mg nail was broken and displaced in the knee joint, which required repair surgery. The remaining patients did not develop postoperative inflammation or new dislocations and healed gradually. This indicates that MAGNEZIX provides sufficient conditions for bone healing. Pure Mg screws can treat femoral head necrosis and have good biocompatibility. Mg screws were implanted in patients with osteonecrosis of the femoral head (ONFH). After 12 months, the Harris Hip Score (HHS) was significantly improved in the Mg screw group, and serum, calcium, magnesium and phosphorus indices were all within normal physiological ranges. The diameter of the Mg screw was reduced by 1.0 ± 0.07 mm after 12 months—a reduction of 25.2 ± 1.8%. The degradation rate was within an acceptable range, and no bone flap displacement or prolapse occurred [91].

### 4.3. Bone Filling

Traditional metal bone implants, such as titanium, stainless steel and cobalt-chromium alloys, have good biocompatibility and impressive mechanical properties. However, these traditional metals cannot biodegrade in the body [6]. Biodegradable Mg alloys make up for this deficiency, and the Mg ions released during the degradation process can promote the mitosis of osteoblasts and enhance bone formation [94]. Therefore, biodegradable Mg alloys have considerable potentials to be used as bone implant materials. Li et al. [95] showed that Mg alloys exerted a good osteogenic effect. Three samples of pure Mg, Mg-3Zn and Mg-2Zn-1Mn were studied. The stimulating effect of these alloys on bone tissue formation was evaluated using a rat femoral fracture model. Mg-2Zn-1Mn alloy showed excellent histocompatibility, stable degradation and a significant osteogenic effect without toxicity. Makkar et al. [96] showed that ZK60 Mg alloy with Ca-Sr-P coating promoted bone formation and can be used for orthopedic implants. The Ca-Sr-P coating promoted adhesion and proliferation of MC3T3-E1 cells. After 4 weeks of implantation in rabbits, the rate of bone formation around the coating was found to be increased. Li et al. [97] showed that biodegradable Mg implants complexed with zoledronic acid (ZA) could treat osteoporosis and bone fracture damage by locally releasing Mg degradation products and zoledronic acid. In vitro studies showed that these implants stimulated the osteogenic differentiation of bone marrow mesenchymal stem cells (rBMSCs) in rats, and ZA inhibited osteoclast generation. After implanting coated Mg intramedullary nails in rats for 12 weeks, under the synergistic action of Mg degradation products and ZA drugs, the bone regeneration rate was considerably increased, the callus was significantly increased, and the quality and mechanical strength of the bone were considerably improved.

### 4.4. Dental Implants

Rider et al. [98] have shown that biodegradable pure Mg (99.95%) barrier membrane guided bone regeneration (GBR) in dental surgery. Mg membranes illustrate the significant advantages of a barrier membrane for GBR treatment. In the initial stage of implantation, the Mg film has a barrier function and a space supply function, in addition to providing a space for the bone graft material in the defect space. During the degradation process of the Mg film, it was gradually covered by new bone; the Mg film ultimately completely degraded, and new tissue appeared. Surface modification is necessary to improve corrosion resistance and bioactivity due to the poor corrosion resistance of Mg film. Peng et al. [99] found that a new calcium phosphate (Ca-P)-coated Mg membrane guided bone regeneration. Ab Mg membrane was implanted into New Zealand white rabbits. The continuously degraded Mg^2+^ guided bone regeneration. The performance of the Mg film was better than that of pure titanium film in the first 4 weeks and better than that of the blank control group within 4–8 weeks before completely degrading after 8 weeks. Ca-P coating improved the corrosion resistance of Mg film by four times. Kacarevic et al. [84] found that Mg-2Y-1Zn-1Mn (WZM211) screws with MgF_2_ coating guided bone regeneration and provided adequate mechanical fixation during healing. Furthermore, 4 weeks after implantation in Yucatan minipigs, the screws largely retained their original shape and volume. The hydrogen produced during the degradation process caused low-level inflammation but did not affect the growth of surrounding alveolar bone tissue. The released Mg^2+^ enhanced the growth of new blood vessels and guided bone regeneration.

### 4.5. Sutures

**Figure 7:**
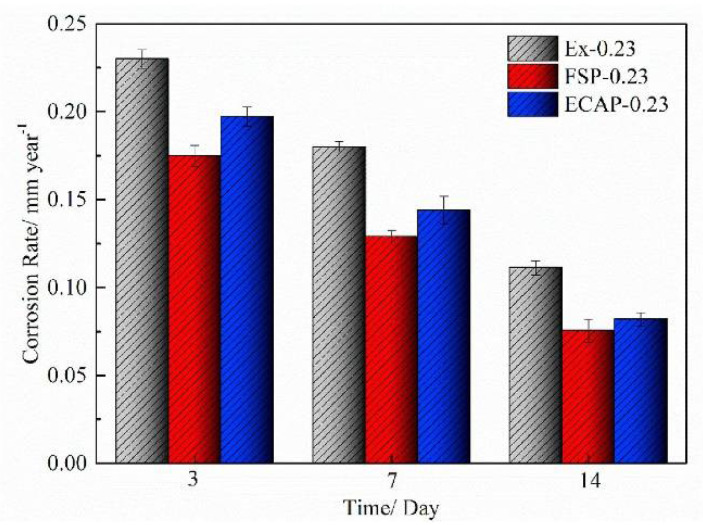
Corrosion rates of ZN20 alloy wires following different processes, calculated from mass loss after immersion in simulated intestinal fluid for 7 days. Ex-0.23: Extrusion-1 and hot drawing; ECAP-0.23: Extrusion-1; ECAP: Extrusion-2 and hot drawing; FSP-0.23: Extrusion-1; FSP: Extrusion-2 and hot drawing. (Reprinted from Ref. [103] with permission).

Sutures play a vital role in the process of tissue repair. The suture should have the following characteristics: (1) good biocompatibility, reducing tissue infection; (2) ease of degradation, reducing the risk of secondary trauma; and (3) a good combination of strength and toughness [100]. Gao et al. [101] showed that Mg-2Zn-0.5Nd (ZN20) alloy filaments could be used as staples in surgery. Fine ZN20 alloy wires were knotted on the intestines and stomachs of New Zealand rabbits. Histological images of HE staining showed that there were no inflammatory cells in the stomachs or intestines. At 8 weeks post implantation, there was no abnormality in the morphology of the gastrointestinal tissue, indicating that the ZN20 alloy had good biocompatibility. The ultimate tensile strength and elongation of fine ZN20 alloy wires were 248 MPa and 13%, respectively, which need to be further improved. The effects of plastic deformation on mechanical properties and corrosion resistance of ZN20 alloy were studied. The results showed that ZN20 alloy with a cumulative area reduction (AR) of 70% after the drawing and annealing processes could further improve its corrosion resistance [102]. Moreover, a combination of channel angular extrusion (ECAP), friction stir machining (FSP), extrusion and hot drawing resulted in an alloy exhibiting the best mechanical properties and corrosion resistance. Figure 7 shows that the corrosion rate of FSP-0.23 was the lowest, indicating that the ZN20 alloy, after combined processing, had good corrosion resistance [103].

## 5. Summary and Outlook

Biodegradable Mg alloys have shown considerable advantages in clinical applications. In this article, the research progresses of high-performance biodegradable Mg alloys, bioactive coatings and actual/possible applications of Mg implants were reviewed. Although there has been a large number of studies on biodegradable Mg alloys, there still exist problems that require further exploration. The summary and outlook of this review are as follows:

1. The biodegradation mechanism of Mg alloys in vivo should be studied further. Because the body environment is very complex, many factors can influence the degradation behavior of Mg alloys, such as protein, body fluids, stress conditions, etc. Only the effects of these factors on the degradation behavior of Mg alloys are well understood, enabling the accurate prediction of the biodegradation of Mg alloys. Researchers can provide timely feedback on the generation of corrosion products around implants through a multifunctional in vivo corrosion characterization system. Additionally, transdermal sensing technology is now being incorporated to monitor the degradation process of such implanted objects.
2. The effects of some elements on the human body should be further illustrated, as the metabolic processes of some of such elements, such as rare earth (RE) metal elements, are not very clear, requiring future systematic studies.
3. Nevertheless, alloying and surface modification are still the main means of improving the mechanical properties and bioactivities of biodegradable Mg alloys. The development of composite coatings is a direction worthy of in-depth research, although their impact on the human body is still relatively unknown. Comparisons and discussions using simulated biological environments (human blood or modified SBF solution) should be further explored to identify the best composite coatings.
4. Many methods are available to evaluate the properties of magnesium alloys. Methods by which to measure the corrosion rate in vitro include weight measurement and electrochemical tests. Moreover, soaking of Mg alloys in human blood or modified SBF solution to observe the antibacterial and cell-proliferation properties of implants are also insightful by which to analyze their biological tendencies. After magnesium alloy products are implanted into the human body, the bone-healing process can be evaluated by X-ray and CT evaluation, and the corrosion rate in the implanted object can be calculated by histological analysis.

## Figures and Tables

**Table 1:** Mechanical properties and corrosion resistance of some typical Mg-RE alloys.

Alloy	YS (MPa)	UTS (MPa)	EL (%)	Corrosion Rate (Weight Loss) (mm/y)	References
Mg-7Y-1.5Nd	165	285	10	19.69	[38]
Mg-2Y-1Zn-0.4Zr-0.3Sr	195	254	27	0.47	[39]
Mg-3Nd-0.2Zn-0.4Zr	293	307	15.9	0.17	[40]
Mg-4Y-2^Nd^-0.8 Zr	221	295	10.7	0.28	[40]
Mg-8.5Gd-5Y-0.2Al	376	263	13	3.8	[41]
Mg-8Y-1Er-2Zn	275	359	19	0.568	[42]

YS, UTS and EL represent yield strength, ultimate tensile strength and elongation, respectively.

## Data Availability

Data sharing is not applicable to this article.
